# Optimization of bovine oocyte cryopreservation: membrane fusion competence and cell death of linoleic acid-*in vitro* matured oocytes subjected to vitrification

**DOI:** 10.3389/fvets.2025.1628947

**Published:** 2025-07-04

**Authors:** Glenda L. Ríos, María Florencia Suqueli García, Rodrigo J. Manrique, Jorgelina Buschiazzo

**Affiliations:** ^1^Laboratorio Biotecnología de la Reproducción, Instituto de Innovación para la Producción Agropecuaria y el Desarrollo Sostenible (IPADS Balcarce), Instituto Nacional de Tecnología Agropecuaria (INTA)-Consejo Nacional de Investigaciones Científicas y Técnicas (CONICET), Balcarce, Argentina; ^2^Facultad de Ciencias Agrarias, Universidad Nacional de Mar del Plata, Balcarce, Argentina

**Keywords:** cryopreservation, linoleic acid, *in vitro* maturation, oocyte, bovine

## Abstract

Long-term conservation of livestock female genetic resources has led to increased interest in oocyte cryopreservation. However, the challenges of preserving large-volume cells and the lack of standardization in vitrification media and cryo-devices for domestic species remain critical areas of ongoing research. This study aimed to assess the impact of *in vitro* maturation medium composition and vitrification devices on cell death and membrane fusion competence of vitrified bovine oocytes, comparing a chemically undefined versus a synthetic maturation medium supplemented with linoleic acid (LA), and using both tubular (open-pulled straw) and surface vitrification devices (Cryotech®). *In vitro* maturation of bovine oocytes under serum-free conditions improved post-vitrification survival, particularly by preserving membrane integrity. Additionally, oocytes matured in a synthetic medium containing epidermal growth factor and hyaluronic acid, and vitrified using a surface device, exhibited increased viability and reduced caspase activation. Supplementation of this medium with 43 μM LA did not compromise oocyte viability or apoptotic status. In contrast, higher concentrations (100 μM) induced significant apoptosis and disrupted membrane integrity, impairing tolerance to cryoprotectant agents. We also identified two distinct localization patterns of the transcription factor OCT4 in matured oocytes, with vitrification promoting the diffuse pattern. Supplementation with LA was insufficient to mitigate the effects of vitrification on OCT4 distribution. Unlike 43 μM, maturation with 100 μM LA increased the proportion of oocytes exhibiting the diffuse OCT4 pattern following exposure to cryoprotectant agents under non-vitrified conditions. Zona *pellucida*-free oocytes co-incubated with sperm demonstrated that 43 μM LA better preserved the original sperm adhesion and fusion pattern post-vitrification, although fertilization rate was not improved. These findings demonstrate that optimized maturation conditions—comprising serum-free media, membrane fluidity-modulating lipids (43 μM LA), and the use of surface cryo-devices—significantly enhance oocyte quality, post-warming survival, and fusion competence, all of which are critical for improving fertilization outcomes following vitrification.

## 1 Introduction

Long-term conservation of livestock female genetic resources reveals a growing interest in oocyte cryopreservation. Just as artificial insemination using frozen semen increases the reproductive efficiency of bulls, oocyte cryopreservation can enhance the reproductive potential of females with high genetic merit. However, oocytes are particularly difficult to cryopreserve mainly due to their large size and consequently low surface-to-volume ratio. In addition, plasma membrane permeability of oocytes at the metaphase II (MII) stage is low, thus making the movement of cryoprotectant agents (CPAs) and water slower (1). Oocytes with a high intracytoplasmic lipid content, such as bovine oocytes, are also more sensitive to chilling (2).

Vitrification is a cryopreservation method characterized by a high concentration of CPAs and a high cooling rate that prevents the formation of ice crystals (3). In this regard, vitrification is the recommended methodology for the cryopreservation of large-volume cells such as oocytes. High cooling rates in vitrification are achieved by directly plunging the samples into liquid nitrogen. Additionally, the combination of different CPAs is used to increase the viscosity of the medium in which the oocytes are suspended, reducing the cytotoxic effect of individual CPAs and favoring vitrification. The probability of vitrification also increases as the volume of the sample decreases (4). Smaller volumes allow better heat transfer, increasing cooling rates and decreasing the probability of fractures of the vitreous solid. To achieve a minimum volume, different devices have been developed. These carrier systems for the manipulation and storage of oocytes or embryos can generally be divided into two categories: tubular devices and surface devices (5). The open-pulled straw (OPS) (6) is a tubing device in which a plastic French mini-straw is heat-softened and pulled manually until the diameter of the central part is reduced by half. The pulled straws are then cut to obtain two OPS. Loading of oocytes or embryos into these straws is performed by capillary touching a 1- to 2-μL drop of vitrification solution containing the oocytes or embryos. On the other hand, Cryotop® is one of the first commercial surface devices in which oocytes or embryos are placed on the top of a very fine polypropylene strip attached to a hard plastic handle (7). If the size of the drop on the strip can be controlled by removing the excess (~0.1 μL), high cooling rate can be achieved. Due to its low cost and good results, the OPS device has been mainly adopted for embryo vitrification in domestic species. Instead, the Cryotop® and other Cryotop-like devices are widely used in the biomedical field. In this scenario, the intrinsic and extrinsic quality of the oocytes also become key factors to obtain an acceptable survival rate for the livestock industry. Bovine oocytes and embryos are often cultured *in vitro* in the presence of fetal bovine serum (FBS). Incubation with FBS not only modifies the lipid composition of bovine oocytes matured *in vitro* (8, 9) but also produces embryos with excessive lipid accumulation and reduced cryotolerance (10). At present, according to international standards and regulations, the use of FBS is being replaced by synthetic serum substitutes (i.e., serum albumin and globulins), recombinant serum albumin and hyaluronic acid (HA) (11). To replace growth factors from FBS, chemically defined cultured media for oocytes tend to incorporate other bioactive maturation inducers such as epidermal growth factor (EGF) family members (12, 13). Previously, we found that bovine oocytes matured in the presence of EGF-HA improved oocyte meiotic competence and embryo developmental timing (9). In addition to oocyte quality, cryosurvival is also determined by the extent of plasma membrane damage and the cell death induced by the cryopreservation procedure (14, 15). Supplementation of culture media with unsaturated fatty acids decreases membrane lipid order by incorporating double bonds into the acyl chains of phospholipids, a strategy previously used to minimize lipid phase transitions during cooling (16, 17). Using radiolabeled linoleic acid (LA), we demonstrated that bovine oocytes are capable of incorporating this polyunsaturated fatty acid from the maturation medium and esterifying it into the main oocyte lipids, such as phospholipids and triglycerides (18). Moreover, esterification of LA in triglycerides stored in lipid droplets revealed a mechanism of oocyte protection against lipotoxicity caused by free fatty acids. Linoleic acid and its conjugated isomers (CLA) have been previously used in culture media to improve bovine oocyte maturation competence and embryo development, taking advantage of their proposed lipid-lowering effects (19, 20). Similarly, the implications of lipid reduction for bovine embryo cryosurvival have been investigated, with most studies focusing on CLA supplementation in culture media (21, 22). However, the role of LA as a membrane fluidity-modulating lipid during oocyte vitrification remains unexplored. The analysis of cell death together with more revealing markers of cell functionality appears to be central to evaluating the effects of cryopreservation under these experimental conditions. In this context, the transcription factor OCT4 is well-known for maintaining pluripotency in the early embryo (23, 24). Interestingly, maternal Oct4 has been identified as a regulator of the developmental competence of the mouse oocyte from oogenesis (25–27). A thorough study combining optimized *in vitro* maturation conditions and minimum-volume devices is still needed to improve the competence of bovine oocytes for cryopreservation and subsequent fertilization.

The main objective of this study was to evaluate the impact of the composition of the *in vitro* maturation medium and vitrification devices on cell death and membrane fusion competence of vitrified bovine oocytes. In particular, we analyzed the effect of a chemically undefined maturation medium and a synthetic one supplemented with LA on oocytes subjected to vitrification, comparing a tubular vitrification device with a surface vitrification device, and making focus on cell death, OCT4 localization, and functional survival, such as the ability of the oocyte’s plasma membrane to successfully fuse with the sperm cell membrane.

## 2 Materials and methods

### 2.1 Chemicals and reagents

All chemicals and reagents were purchased from Sigma-Aldrich/ Merck Chemical Company, unless otherwise stated.

### 2.2 Oocyte collection and *in vitro* maturation

Bovine ovaries from cycling beef heifers (*Bos taurus taurus*) were collected from local slaughterhouses and transported within 60 min of harvest in a thermo-regulated container to the laboratory. *Cumulus-*oocyte complexes (COCs) were aspirated from follicles ranging from 2 to 8 mm in diameter by a *vacuum* system. *Cumulus-*oocyte complexes with homogeneous ooplasm and more than four complete layers of *cumulus* cells, corresponding to grades 1 and 2 according to de Loos et al. (28), were selected under a stereomicroscope and washed 3 times in phenol red-free HEPES-buffered Synthetic Oviductal Fluid (HSOF) supplemented with 1% polyvinyl alcohol (PVA) (w/v). Selected COCs were randomly divided into two different experimental groups according to the composition of the maturation media. For both groups, the base medium consisted of M199 plus 0.1 mg/mL L-glutamine and 2.2 mg/mL NaHCO_3_. The first experimental group (FSH-FBS) was supplemented with 0.01 IU/mL rh-FSH (Gonal F-75, Serone, UK) and 10% FBS, and the second group (EGF-HA) with 10 ng/mL EGF, 15 μg/mL HA, and 100 μM cysteamine as anti-oxidant. *Cumulus-*oocyte complexes were placed in four-well dishes (NUNC, Thermo Fisher Scientific, UK) in 400 μL of maturation medium and incubated in groups up to 60 COCs per well for 22 h at 38.5°C under 5% CO_2_ in humidified air. Additionally, COCs from EGF-HA group were *in vitro* matured in the presence of 43 μM or 100 μM LA (18). A commercial water-soluble version of bovine serum albumin (BSA)-conjugated LA was used. Before CPA-exposure or vitrification, selected COCs matured *in vitro* were partially denuded of *cumulus* cells by gentle pipetting in HSOF-PVA.

### 2.3 Vitrification and warming

#### 2.3.1 Tubular device: OPS

Vitrification was performed as previously described by Vajta et al. (6). The holding medium used for handling oocytes during vitrification and warming was phenol red- and calcium-free HSOF-PVA with protein supplement. All manipulations were performed on a 39°C heated stage. Media were used at room temperature, except for the warming solution which was used at 37°C. Partially denuded oocytes were suspended in equilibration solution containing 7.5% ethylene glycol (EG) (v/v) and 7.5% dimethyl sulfoxide (DMSO) (v/v) for 5 min. Following equilibration, they were transferred to the vitrification solution containing 16.5% EG (v/v), 16.5% DMSO (v/v) and 0.5 M sucrose, for 45–60 s. Loading of partially denuded COCs into the OPS was performed by capillary from a 1- to 2-μL drop. After loading, the OPS was immediately plunged into liquid nitrogen. For warming, the OPS end with the oocyte was directly submerged in warming solution containing 0.25 M sucrose for 1 min. The warmed-oocytes were then transferred to a solution containing 0.15 M sucrose for 5 min, and then washed in HSOF-PVA for 5 min. Recovery of COCs was achieved at the incubator for 2 h. When OPS was compared with the surface device Cryotech®, the same protocol of vitrification and warming was followed (14).

#### 2.3.2 Surface device: Cryotech®

Vitrification was performed following the protocol of Zhou et al. (29) using vitrification and warming solutions of known composition (14). These protein free solutions were adapted from the literature (29, 30) replacing serum by the synthetic polymer PVA. Phenol red- and calcium-free HSOF-PVA was used to handle COCs and as base medium to prepare vitrification and warming solutions (30). Manipulations were performed on a 39°C heated stage and vitrification solutions were maintained at room temperature (25–27°C). Partially *cumulus*-denuded oocytes (2 *cumulus* cell layers) were equilibrated for 10 min in equilibration solution with 7.5% EG (v/v) and 7.5% DMSO (v/v) and vitrified in vitrification solution with 15% EG (v/v), 15% DMSO (v/v) and 0.5 M sucrose for 45–60 s including mounting onto Cryotech® device (2–3 oocytes/device) and plunging into liquid nitrogen. Almost all vitrification solution on the device was removed to leave only a thin layer covering the COCs. For warming, Cryotech® device was directly submerged into 1 M sucrose solution for 1 min and then transferred into 0.5 M sucrose solution for 3 min. Subsequently, oocytes were washed twice for 5 min in HSOF-PVA. The heated stage was used at 39°C and warming solutions were maintained at 37°C during the whole procedure. Recovery of COCs was achieved at the incubator for 2 h.

### 2.4 *In vitro* fertilization of *zona pellucida*-free oocytes

*In vitro* fertilization of *zona pellucida* (ZP)-free oocytes was performed by adapting protocols followed for murine (31) and bovine oocytes (32). *Cumulus* cells were removed by a brief exposure to hyaluronidase (1 mg/mL). Only oocytes showing a visible polar body after *in vitro* maturation were used in this assay. The ZP was digested with 1 mg/mL pronase (protease from *Streptomyces griseus*) under visual monitoring. The ZP-free oocytes were rapidly washed in HSOF-PVA and kept at 38.5°C under 5% CO_2_ in humidified air for 2 recovery h. Next, ZP-free oocytes were inseminated in 100 μL drops of IVF-SOF medium supplemented with 12 μg/mL heparin. Co-incubation with sperm at a final concentration of 1 × 10^5^ spermatozoa/mL was performed for 16 h at 38.5°C under 5% CO_2_ in humidified air. After insemination, the oocytes were washed through 3 drops by the same person using the same thin-bore pipette. Oocytes were then stained with 5 μM bisBenzimide Hoechst 33342 for 5 min at room temperature and mounted for analysis under an epifluorescence microscope (Nikon TE-300; Nikon, Tokyo, Japan). Digital photographs were taken with a DSfi1 camera (Nikon, Tokyo, Japan) connected to the microscope.

### 2.5 Annexin V staining of externalized PS

Exposure of phosphatidylserine (PS) on the outside bilayer of the plasma membrane of apoptotic oocytes was detected by a fluorescent version of Annexin V, a protein with high affinity for PS (33). Oocytes were denuded of *cumulus* cells by pipetting in 1 mg/mL hyaluronidase for 1–5 min. Non-fixed living oocytes were then transferred to 500 μL of 1X Binding Buffer (Annexin V-FITC Apoptosis Detection Kit, Calbiochem®) and 1.25 μL Annexin V-FITC, and incubated for 15 min at room temperature in the dark. After incubation, oocytes were washed three times in HSOF-PVA with 1:500 propidium iodide (PI) (Calbiochem®) and then mounted on slides. The oocytes were observed using an excitation wavelength of 450–490 nm in an epifluorescence inverted microscope (Nikon TE-300; Nikon, Tokyo, Japan) and imaged with a DSfi1 camera (Nikon, Tokyo, Japan) connected to the microscope. A positive control of oocytes exposed to UV light was included in each trial. Oocytes were classified as viable oocytes, with no Annexin V and no PI staining (A−/PI−); apoptotic oocytes with homogeneous Annexin V green bright signal on the plasma membrane, but PI negative (A+/PI−); and non-viable oocytes, showing or not Annexin V staining on the membrane together with PI red fluorescence inside the oocyte (A+/PI+ or A-/PI+) (34). Unlike other cell types that concentrate PI fluorescence in the cell nucleus (intercalation with DNA), MII oocytes with no nuclear envelope also show PI red fluorescence in the cytoplasm.

### 2.6 *In situ* detection of activated caspases

Apoptosis was analyzed in living cells through detection of activated caspases with a specific fluorescent inhibitor (VAD-FMK-FITC, Calbiochem®). Caspase inhibitor (VAD-FMK) conjugated to FITC is cell permeable, nontoxic, and irreversibly binds to activated caspases in apoptotic cells. *Cumulus* cell-denuded oocytes were incubated in 1:300 VAD-FMK-FITC in M199 for 45 min at 38.5°C in the dark under 5% CO_2_ in humidified air. Oocytes were then washed in HSOF-PVA with 1:500 PI (Calbiochem®) to assess membrane integrity. They were finally mounted for detection by fluorescence microscopy with an excitation wavelength of 450–490 nm microscope Nikon TE-300; Nikon, Tokyo, Japan. Oocytes were classified into 3 groups: viable oocytes, with a basal green signal (C-), apoptotic caspase-positive oocytes, showing brilliant green fluorescence (C+), and non-viable oocytes, with red/orange fluorescence (C+ PI+).

### 2.7 Immunofluorescence detection and localization of OCT4

Detection and subcellular localization of OCT4 was evaluated by immunofluorescence in matured oocytes (MII), under different experimental conditions: in fresh (F) and after vitrification-warming (V). *Cumulus* cells and ZP were removed as previously described. ZP-free oocytes were fixed in 2% paraformaldehyde at room temperature for 20 min. Fixed oocytes were then permeabilized in 0.3% Triton X-100 at room temperature for 10 min and immediately washed for 5 min in PBS supplemented with 0.1% BSA. Blocking was performed at room temperature in PBS supplemented with 1% BSA for 1 h. Oocytes were incubated overnight in 1:25 (v/v) anti-Oct4 ^3/4^ (N19 polyclonal goat antibody sc-8628, Santa Cruz Biotechnologies, CA) in PBS/1% BSA at 4°C. Incubation with the secondary antibody anti-goat Alexa Fluor^555^ (Thermo Fisher) in a dilution 1:600 (v/v) was performed for 1 h at room temperature in the dark. Negative controls were performed avoiding primary antibody. The location of the metaphase plate was identified by Hoechst 33342 staining. Finally, oocytes were mounted in ProLong™ Gold Antifade Mountant medium (Thermo Fisher) for observation by fluorescence microscopy (microscope Nikon TE-300; Nikon, Tokyo, Japan) and imaged with a DSfi1 camera (Nikon, Tokyo, Japan) connected to the microscope.

### 2.8 Statistical analysis

Statistical analysis was conducted using INFOSTAT and R 4.2.2 software (35, 36). Binomial distribution variables were compared using General Linear Models. The *post-hoc* Fisher’s LSD test was applied when ANOVA revealed significant effects for the analyzed variables (*p* < 0.05). All experiments were repeated at least three times. The percentages are expressed as means ± SEM. The number of repetitions and oocytes for each evaluation were indicated in the corresponding figure legend or table.

## 3 Results

### 3.1 Externalization of PS in CPA-exposed or vitrified oocytes matured *in vitro* with FSH-FBS or EGF-HA

**Figure 1:**
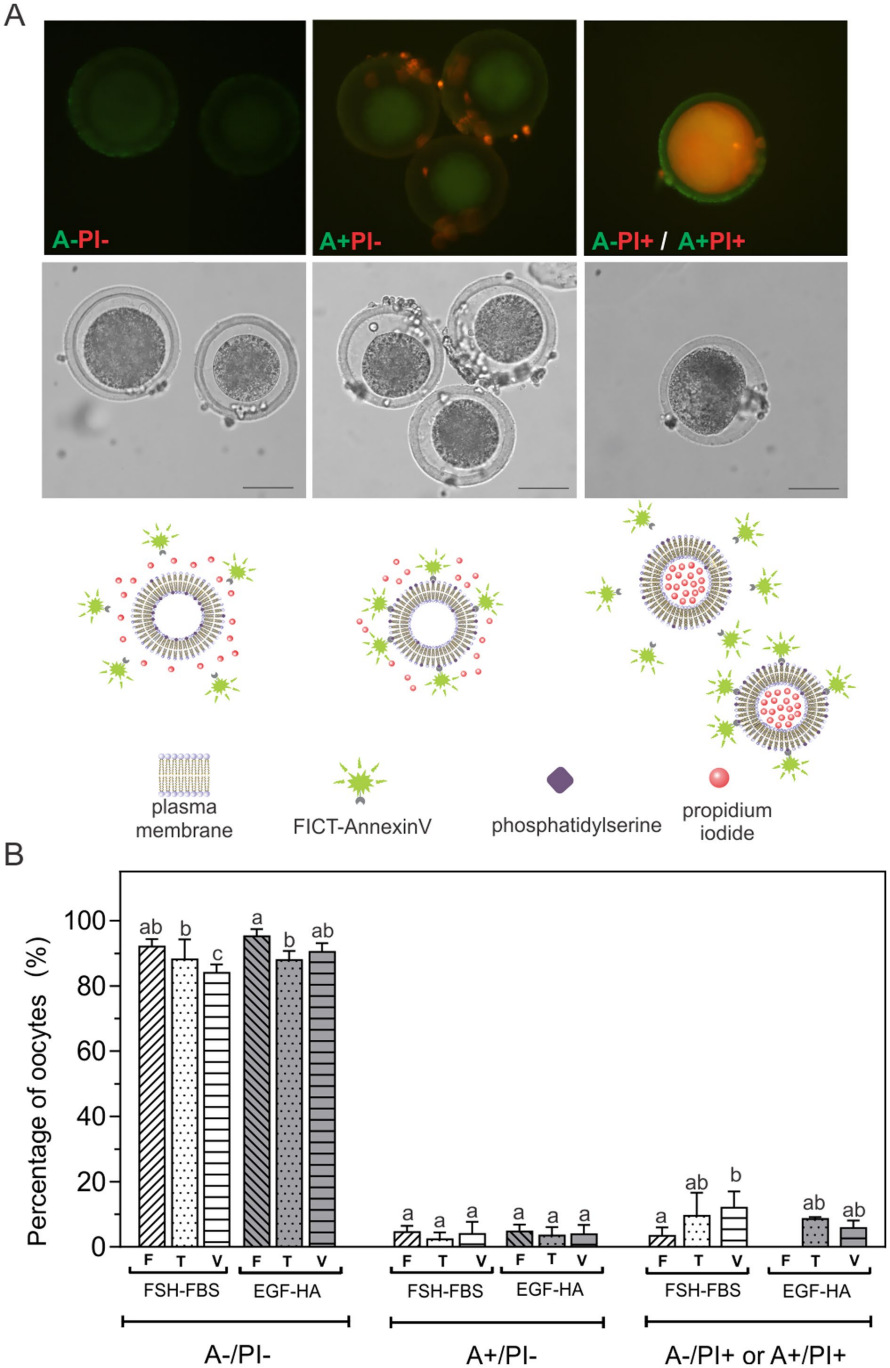
Externalization of PS after exposition to equilibrium, vitrification and warming solutions or vitrification of oocytes *in vitro* matured in FSH-FSB or EGF-HA. **(A)** Fluorescent patterns of bovine oocytes assessed by FITC-Annexin V/PI staining, their respective bright field images (middle) and schematic diagrams of the fluorescent patterns (below). **(B)** Percentages of oocytes with different FITC-Annexin V/PI fluorescent patterns. Results represent mean values ± SEM from 3 independent experiments. Fresh oocytes (F) *in vitro* matured in FSH-FBS (*n* = 88) or in EGF-HA (*n* = 105); oocytes exposed to CPAs, referred to as the toxicity group (T), *in vitro* matured in FSH-FBS (*n* = 62) or in EGF-HA (*n* = 68); vitrified-warmed oocytes (V) *in vitro* matured in FSH-FBS (*n* = 63) or in EGF-HA (*n* = 62). Different letters (a–c) indicate significant differences (*p* < 0.05) among experimental conditions within each apoptosis status. For vitrified oocytes, the OPS cryo-device was used following the protocol of Vajta et al. (6). A-/PI-: viable oocytes; A+/PI-: apoptotic oocytes with surface-exposed PS; A-/PI+ or A+/PI+: non-viable oocytes. Remaining PI+ *cumulus* cells are observed in A+/PI- panel (Magnification 40X). Scale Bar: 50 μm.

In order to evaluate whether the composition of the *in vitro* maturation medium affects oocyte quality in terms of apoptotic status, we analyzed PS externalization at the outer leaflet of the plasma membrane of oocytes exposed to equilibrium, vitrification and warming solutions, referred to as toxicity (T) caused by CPA-exposure. These oocytes were not plunged into liquid nitrogen. We also evaluated how the oocytes respond to the vitrification process. Therefore, oocytes were subjected to vitrification (V) following the protocol of Vajta et al. (6), using OPS as the vitrification device. Annexin V (A) assay to assess PS externalization was performed by incubation of non-fixed living cells in combination with PI (dual staining). Oocytes exhibited three fluorescence patterns (Figure 1A): A-/PI- (viable oocytes); A+/PI- (apoptotic oocytes with surface-exposed PS), and A+/PI+ or A-/PI+ (non-viable oocytes). Vitrification (V) decreased viable oocytes (A-/PI-) (*p* < 0.05), particularly when the oocytes were matured in FSH-FBS (Figure 1B). The level of oocytes with exposed PS (A+/PI-) was relatively low (< 5%) and did not show differences among experimental conditions, regardless of the maturation medium used or the treatment applied (CPA-exposure or vitrification). However, vitrification significantly increased non-viable oocytes (A+/PI+ or A-/PI+) in the FSH-FBS group (Figure 1B). This effect was not evidenced in oocytes matured in EGF-HA. Moreover, fresh oocytes matured in EGF-HA did not show PI+ oocytes.

### 3.2 Activation of caspases in CPA-exposed or vitrified oocytes matured *in vitro* with FSH-FBS or EGF-HA

**Figure 2:**
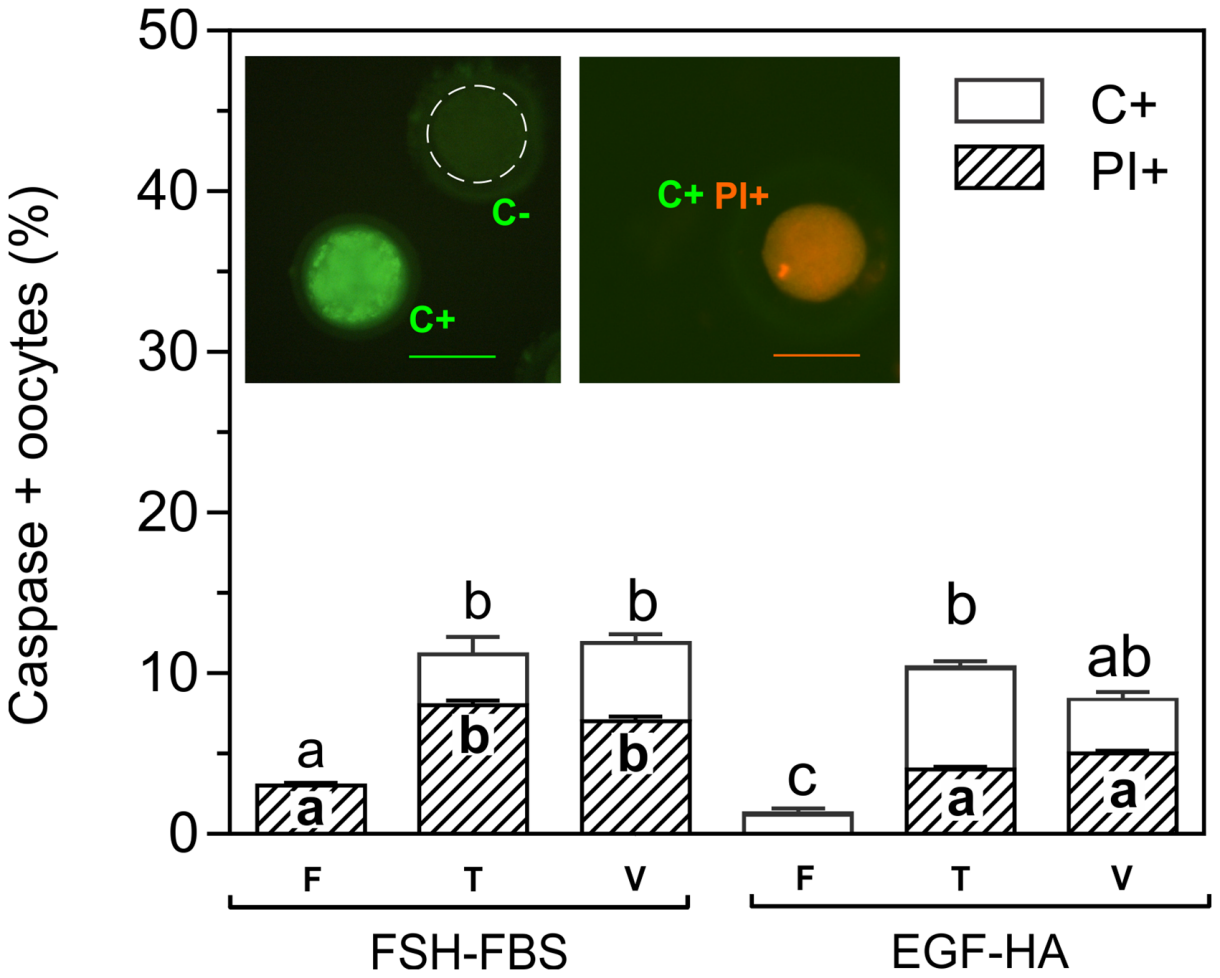
Effect of exposure to CPAs or vitrification on caspase activation and membrane integrity of oocytes matured with FSH-FBS or EGF-HA. Results are shown as mean values ± SEM expressed as percentages of caspase-positive (C+) and PI+ oocytes from 4 independent experiments. Fresh oocytes (F) *in vitro* matured in FSH-FBS (*n* = 125) or EGF-HA (*n* = 106); oocytes exposed to CPAs, referred to as the toxicity group (T), *in vitro* matured in FSH-FBS (*n* = 83) or EGF-HA (*n* = 143); vitrified-warmed (V) oocytes *in vitro* matured in FSH-FBS (*n* = 82) or EGF-HA (*n* = 105). Different letters (a–c) indicate significant differences (*p* < 0.05) among experimental conditions. For vitrified oocytes, the OPS cryo-device was used following the protocol of Vajta et al. (6). Oocytes C+ (brilliant green fluorescence), caspase-negative (C-) (dashed line), and C+/PI+ (orange fluorescence) are shown in the image inserts. Magnification 20X. Scale bar: 100 μm.

Detection of activated caspases was analyzed in non-fixed living cells by a fluorescent specific inhibitor (FITC-VAD-FMK). This permeable fluorescent probe binds irreversibly to activated caspases in apoptotic oocytes allowing direct detection by fluorescence microscopy. Caspase positive oocytes showed brilliant green fluorescence (Figure 2, insert). This assay was carried out in the presence of PI to assess possible alterations in membrane integrity. Activation of caspases was analyzed in fresh oocytes and in oocytes exposed to CPAs (T) or vitrified (V) with OPS (tubular device), following the protocol of Vajta et al. (6). Basal caspase activation (~2%) was found in fresh oocytes (F), particularly in oocytes matured in EGF-HA (Figure 2). No statistical differences in the percentage of C+ oocytes were found between maturation media after CPA-exposure (T) or vitrification (V). Exposure to CPAs and vitrification similarly increased activation of caspases (~12%) in both maturation groups (FSH-FBS and EGF-HA) with respect to their respective fresh condition, except in the vitrified oocytes matured in EGF-HA. This experimental group showed intermediate values thus, no statistical differences were found with respect to the respective toxicity condition or the fresh FSH-FBS group (Figure 2). Membrane integrity assessed by PI staining showed increased levels of PI+ oocytes when oocytes were matured in the presence of serum (FSH-FBS group) (Figure 2), both in fresh oocytes and after exposure to CPAs or vitrification. Unlike oocytes matured in FSH-FBS, fresh oocytes matured in EGF-HA did not show PI+ oocytes. Interestingly, all the oocytes PI+ were also C+, regardless of the experimental condition analyzed.

### 3.3 Activation of caspases in oocytes vitrified with OPS or Cryotech® devices

**Figure 3:**
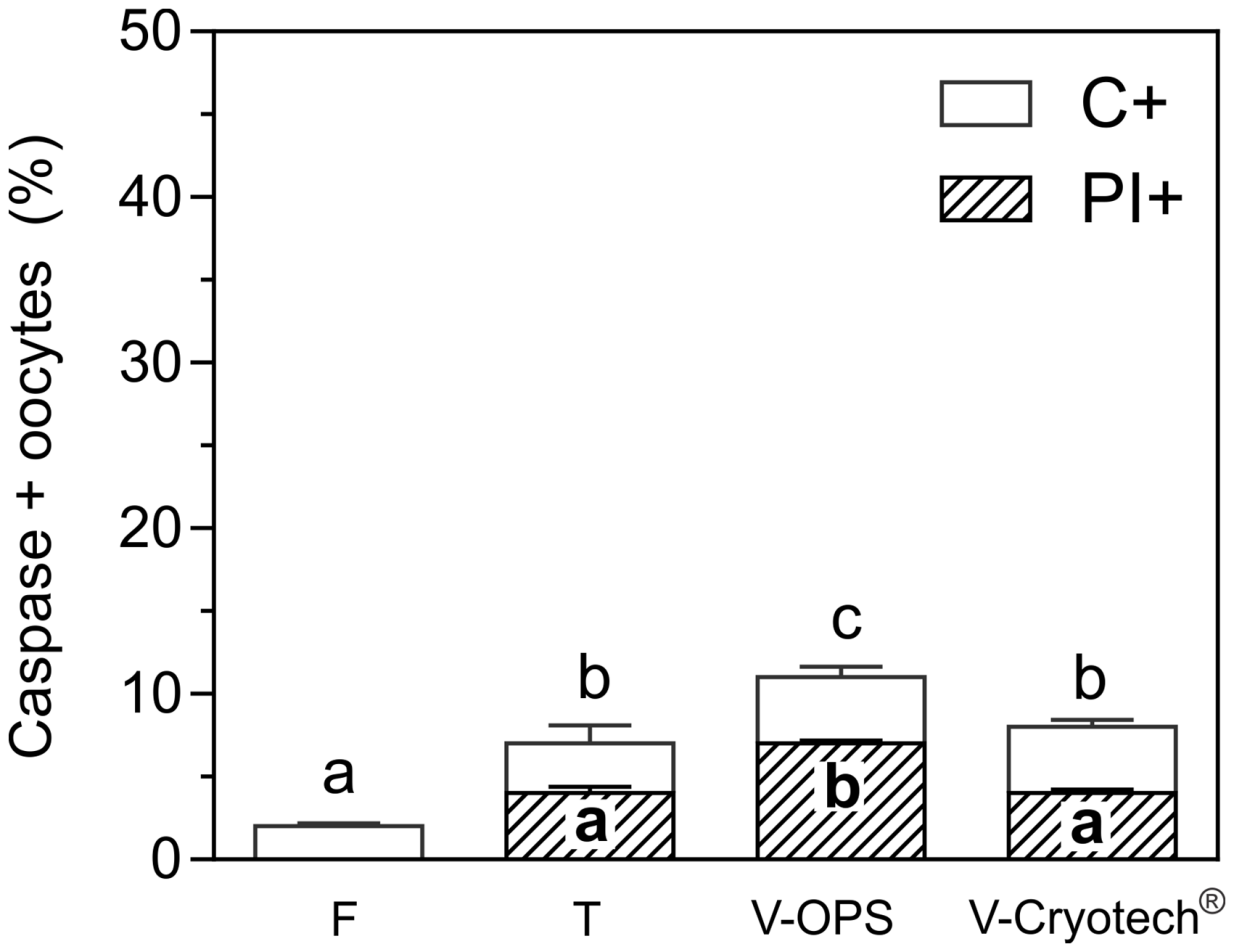
Activation of caspases and membrane integrity in oocytes matured with EFG-HA and vitrified using OPS or Cryotech®. To compare both devices, the same protocol of vitrification and warming was followed (14). Oocytes were incubated with FITC-VAD-FMK to detect *in situ* activated caspases and subsequently incubated with PI to assess membrane integrity. Results are shown as mean values ± SEM expressed as percentages of caspase-positive oocytes (C+) or C+ and PI+ (C+/PI+) oocytes from 4 independent experiments. Different letters (a–c) indicate significant differences (*p* < 0.05) among experimental conditions. Fresh oocytes (F) (*n* = 83); oocytes exposed to CPAs, referred to as the toxicity group (T) (*n* = 92); oocytes vitrified (V) in OPS (*n* = 74) or Cryotech® (*n* = 83).

Compared to oocytes matured in FSH-FBS, the oocytes matured in EGF-HA revealed a slightly better response to vitrification in terms of Annexin V/PI and caspase activation. Therefore, the effect of two different vitrification devices on EGF-HA-matured oocytes was then evaluated. To compare a tubular vitrification device (OPS) and a surface vitrification device (Cryotech®), the same protocol of vitrification and warming was followed (14). Oocytes were incubated in FITC-VAD-FMK together with PI to detect *in situ* activated caspases and to assess membrane integrity, respectively. The level of caspase activation increased after CPA-exposure or vitrification (*p* < 0.05), particularly when oocytes were vitrified in OPS (Figure 3). On the other hand, the level of caspase activation of oocytes vitrified using Cryotech® did not differ from that observed in the toxicity group (Figure 3).

As to membrane integrity, CPA-exposure and vitrification with Cryotech® similarly increased the level of PI+ oocytes compared to the fresh control (Figure 3). In contrast, oocytes vitrified in OPS showed higher percentages of PI+ oocytes compared to the other experimental groups. As previously observed (Figure 2), all the oocytes PI+ were also C+ .

### 3.4 Activation of caspases in oocytes matured in the presence of LA and vitrified with the surface device Cryotech®

**Figure 4:**
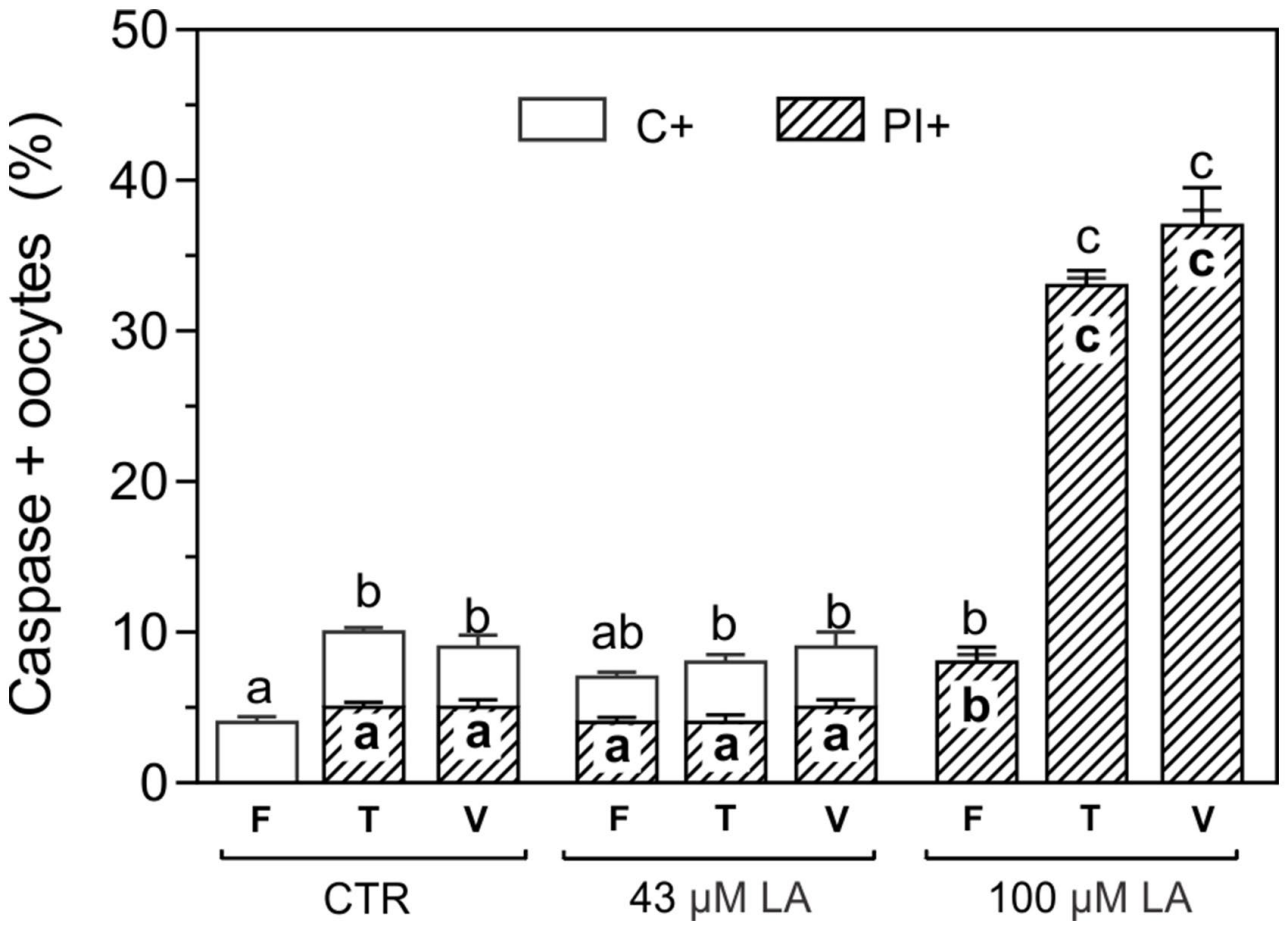
Activation of caspases in oocytes matured with EFG-HA in the presence of LA at two concentrations (43 μM and 100 μM) and vitrified using a Cryotech® device. Oocytes were vitrified following a previously described protocol (14). Results are shown as mean values ± SEM expressed as percentages of caspase-positive (C+) and PI+ oocytes from 3 independent experiments. Different letters (a–c) indicate significant differences (*p* < 0.05) among experimental conditions. Fresh oocytes (F): CTR (*n* = 64); 43 μM LA (*n* = 66); 100 μM LA (*n* = 66). Oocytes exposed to CPAs, referred to as the toxicity group (T): CTR (*n* = 84); 43 μM LA (*n* = 53); 100 μM LA (*n* = 54). Vitrified oocytes (V) using a Cryotech® device: CTR (*n* = 56); 43 μM LA (*n* = 52); 100 μM LA (*n* = 60).

In order to evaluate the possible lipotoxicity generated by free fatty acids, oocytes matured with EGF-HA in the presence of 43 μM or 100 μM LA were exposed to CPAs (T) or vitrified (V) (14) using a Cryotech® device, and subsequently incubated in FITC-VAD-FMK/PI to assess caspase activation and membrane integrity, respectively (Figure 4). After exposure to CPAs or vitrification similar levels of C+ oocytes were found in oocytes matured in the absence or presence of 43 μM LA (Figure 4). As to membrane integrity, ~ 4% of the fresh oocytes matured with 43 μM LA were PI+ but this level was maintained after CPA-exposure or vitrification. On the other hand, fresh oocytes *in vitro* matured in the presence of 100 μM LA showed significantly higher percentages of PI+ oocytes (8%) compared to the other experimental conditions (CTR or 43 μM LA), even exceeding the values reached after exposure to CPAs or vitrification in these groups. After CPA-exposure or vitrification, both C+ and PI+ oocytes significantly increased in oocytes matured in 100 μM LA.

### 3.5 OCT4 localization patterns in oocytes matured in the presence of LA and vitrified using a Cryotech® device

**Figure 5:**
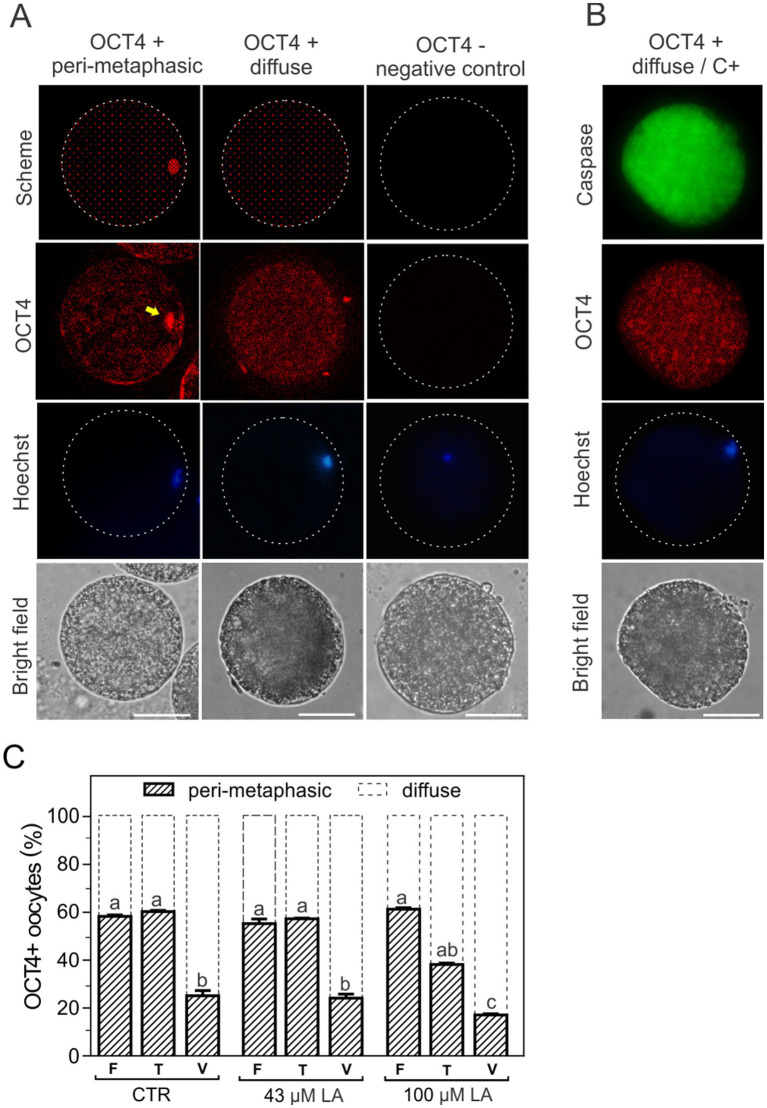
Patterns of OCT4 fluorescence localization in bovine oocytes matured *in vitro*. **(A)** OCT4 fluorescence showed two types of subcellular localization, peri-metaphasic and diffuse (schematized in the first panel and visualized in the second panel). The location of the metaphase plate was identified by Hoechst 33342 staining (third panel). The last panel shows the bright field. Magnification 40X, scale bar: 50 μm. A negative control of OCT4 was obtained by incubation in the absence of primary antibody (third column). **(B)** Activation of caspases (C+) and OCT4 localization pattern after oocyte vitrification. **(C)** Distribution of OCT4 patterns after CPA-exposure or vitrification of oocytes matured in the absence or presence of LA at 43 μM or 100 μM. Results are expressed as percentage of oocytes for each OCT4 pattern in the different experimental groups. Control oocytes (CTR): fresh (F) (*n* = 31); toxicity (T) (*n* = 30); vitrified (V) (*n* = 36). Oocytes matured in 43 μM LA: fresh (F) (*n* = 25); toxicity (T) (*n* = 33); vitrified (V) (*n* = 31). Oocytes matured in 100 μM LA: fresh (F) (*n* = 33); toxicity (T) (*n* = 25); vitrified (V) (*n* = 34). The toxicity group (T) comprises the oocytes exposed to CPAs. For all maturation conditions, oocytes were vitrified using a Cryotech® device, following a previously described protocol (14).

In order to identify the subcellular localization of the transcription factor OCT4, oocytes matured in the presence of 43 μM or 100 μM LA were exposed to CPAs (T) or vitrified (V) (14) using the Cryotech® device and subsequently incubated with the corresponding antibodies to visualize OCT4 by immunofluorescence. Oocytes were also incubated with Hoechst 33342 for DNA labeling and thus localization of the oocyte metaphase plate. Immunofluorescence revealed two patterns of OCT4 localization: peri-metaphasic and diffuse (Figure 5A). Oocytes with peri-metaphasic localization of OCT4 (Figure 5A, yellow arrow second panel) showed intense fluorescence near the metaphase plate, as pointed out by Hoechst staining (Figure 5A, third panel), and relatively moderate, heterogeneous fluorescence in the cytoplasm. Otherwise, OCT4-positive oocytes showed a diffuse homogeneous fluorescence throughout the whole cytoplasm (Figure 5A, second panel). No fluorescence was observed in negative control experiments omitting the primary antibody (Figure 5A, third column). Interestingly, when caspase activation was evaluated after inducing apoptosis by vitrification, all C+ oocytes exhibited the diffuse pattern (Figure 5B). Fresh oocytes for all experimental groups showed a relatively even percentage distribution between the peri-metaphasic and diffuse patterns (60–40%, respectively), with more representation of the peri-metaphasic pattern. Vitrification significantly modified the distribution between OCT4 patterns, increasing the proportion of oocytes with diffuse signal (~80%), regardless of the presence or not of LA during maturation (Figure 5C). However, toxicity monitoring when oocytes were matured in the presence of 100 μM LA revealed a decrease of more than 20% (*p* < 0.05) in the peri-metaphasic pattern with respect to the fresh control, with a concomitant increase in the diffuse pattern (Figure 5C). At this high concentration of LA, the patterns peri-metaphasic and diffuse of OCT4 were inverted (~40–60%, respectively) compared to the corresponding fresh control (60–40%, respectively).

### 3.6 Sperm adhesion and fusion to ZP-free oocytes matured in the presence of increasing concentrations of LA

*In vitro* binding and fertilization assays were performed by insemination of ZP-free oocytes matured in the presence of 43 μM or 100 μM LA. Fertilization was assessed by registering the number of Hoechst-labeled decondensed sperm heads per oocyte. Oocytes containing at least one decondensed sperm head in their cytoplasm were considered fertilized. The oocytes were classified in ranges based on the number of decondensed sperm heads per oocyte: 0 (unfertilized), 1–3, 4–6, 7–9 and ≥10 decondensed sperm heads. Simultaneously, the number of sperm bound to the plasma membrane of each oocyte was evaluated and the most frequent value (mode) was established for each range of fertilized oocytes. Unfertilized oocytes without any bound sperm were presumed to be dysfunctional and, therefore, excluded from subsequent analysis.

**Figure 6:**
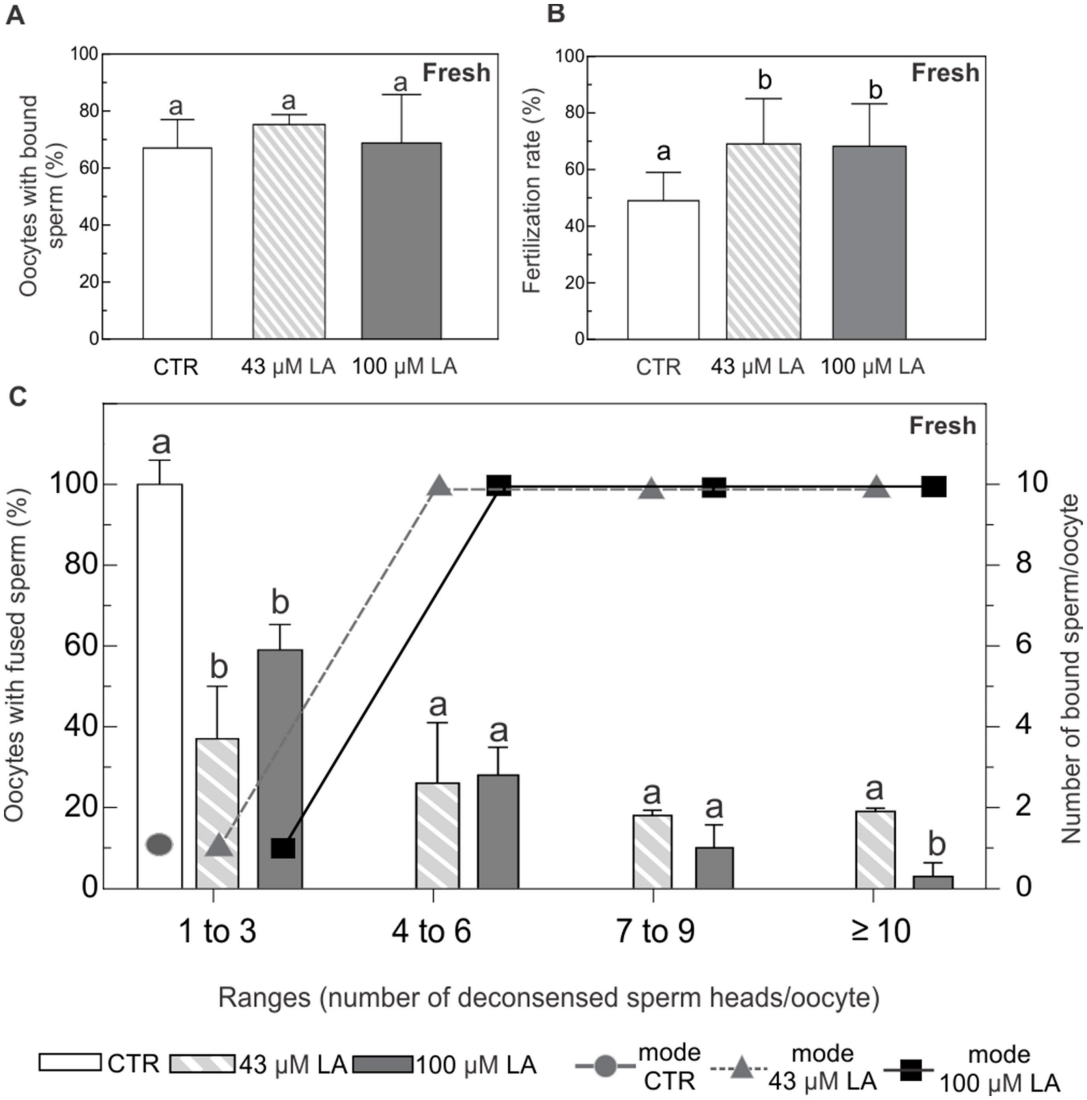
Binding and fusion of Hoechst-labeled sperm to ZP-free oocytes matured in the presence of 43 μM or 100 μM LA. **(A)** Percentage of ZP-free MII oocytes with bound sperm. **(B)** Fertilization rate expressed as percentage of fertilized ZP-free MII oocytes. **(C)** Percentage of oocytes with fused spermatozoa and the most frequent number (mode) of sperm bound per oocyte, analyzed in ranges based on the number of decondensed sperm heads per oocyte (1–3, 4–6, 7–9 and ≥10). Fresh ZP-free MII oocytes *in vitro* matured in the presence of 43 μM or 100 μM LA were inseminated with 10^5^ sperm/mL for 16 h. Sperm fusion and binding were observed by fluorescent microscopy after Hoechst staining. Results represent mean values ± SEM from 3 independent experiments. Control oocytes (CTR) (*n* = 52); oocytes matured in 43 μM LA (*n* = 62); oocytes matured in 100 μM LA (*n* = 59). Different letters (a,b) indicate significant differences (*p* < 0.05) among experimental conditions within each range.

*In vitro* maturation in the presence of LA did not affect oocyte plasma membrane ability to bind sperm. In all experimental conditions, including the control (−LA), ~70% of the oocytes showed sperm adhered to their plasma membrane (Figure 6A). Compared to the control condition, fertilization rate was statistically higher in the oocytes matured in the presence of LA, regardless of the LA concentration (Figure 6B).

All the oocytes in the control group showed between 1 and 3 fused spermatozoa with nuclear decondensation. In addition, the most frequent case observed was that of a single spermatozoon adhered to the plasma membrane of the oocyte (Figure 6C). Although *in vitro* maturation with LA at both concentrations similarly decreased the percentage of oocytes with 1 to 3 fused sperm, it did not affect the most frequent number of membrane-bound sperm at this range of fertilized oocytes (Figure 6C). Oocytes matured *in vitro* in the presence of LA, regardless of the concentration used, showed a higher number of fused sperm covering all ranges. These oocytes showed a markedly increased number of membrane-bound spermatozoa.

**Figure 7:**
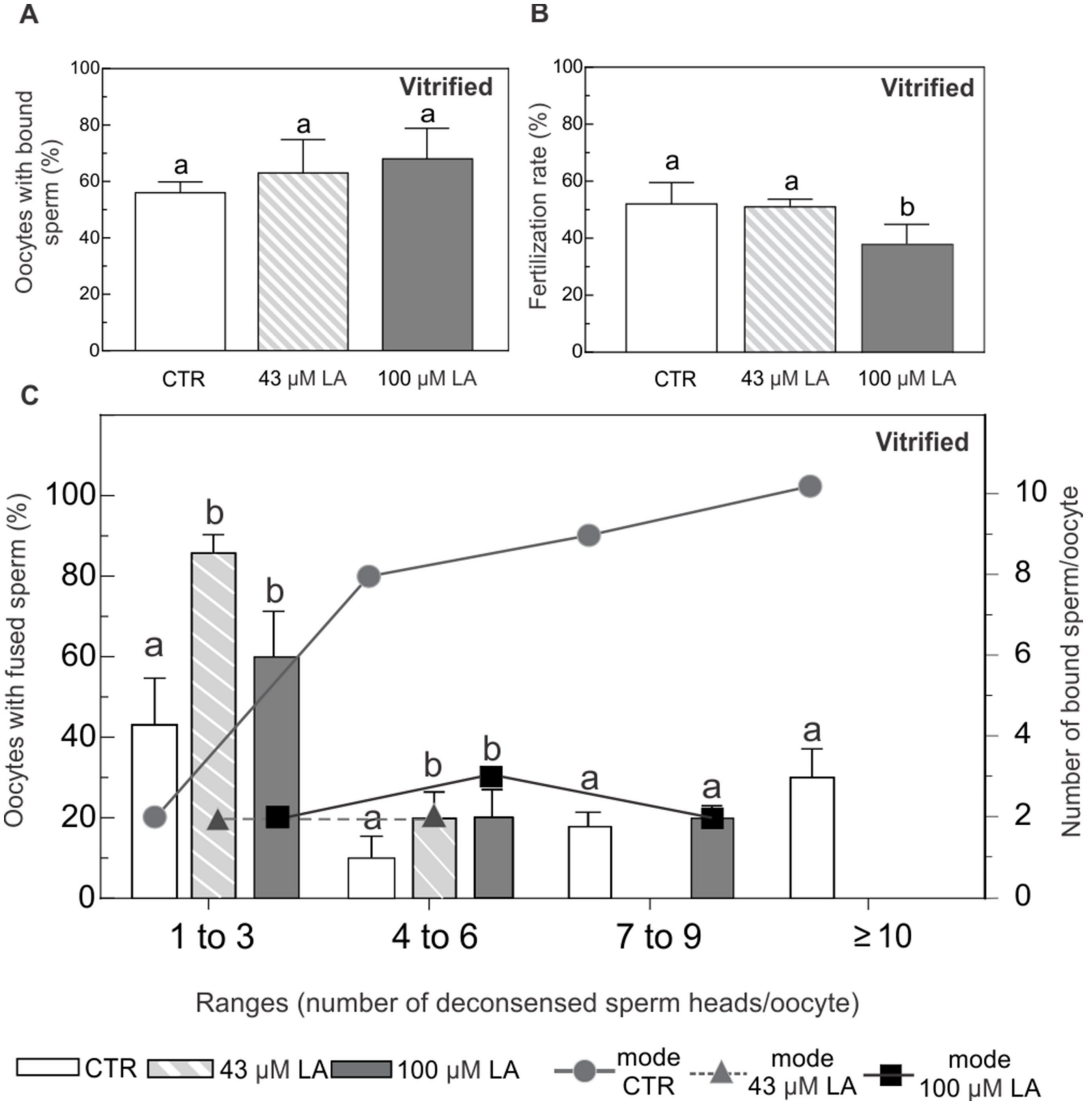
Binding and fusion of Hoechst-labeled sperm to ZP-free oocytes matured in the presence of 43 μM or 100 μM LA and vitrified using a surface device. **(A)** Percentage of ZP-free MII oocytes with bound sperm after vitrification. **(B)** Fertilization rate expressed as percentage of fertilized ZP-free MII oocytes after vitrification. **(C)** Percentage of oocytes with fused spermatozoa and the most frequent number (mode) of sperm bound per oocyte, analyzed in ranges based on the number of decondensed sperm heads per oocyte (1–3, 4–6, 7–9 and ≥10), after vitrification. Vitrified-warmed ZP-free MII oocytes *in vitro* matured in the presence of 43 μM or 100 μM LA were inseminated with 10^5^ sperm/mL for 16 h. Sperm fusion and binding were observed by fluorescent microscopy after Hoechst staining. For all maturation conditions, oocytes were vitrified using a Cryotech® device, following a previously described protocol (14). Results represent mean values ± SEM from 3 independent experiments. Control oocytes (CTR) (*n* = 46); oocytes matured in 43 μM LA (*n* = 45); oocytes matured in 100 μM LA (*n* = 47). Different letters (a,b) indicate significant differences (*p* < 0.05) among experimental conditions within each range.

After vitrification, ~60–70% of the oocytes showed adhered sperm to their plasma membrane, regardless of the experimental condition (Figure 7A). With respect to fertilization rate, oocytes matured in the presence of 43 μM LA showed similar fertilization rate, compared to the vitrified control group (Figure 7B). Conversely, oocytes matured in 100 μM LA exhibited a lower fertilization rate with respect to control and 43 μM LA groups.

Regardless of the presence and concentration of LA, after vitrification oocytes distributed in all the ranges of fused sperm (Figure 7C). Control oocytes showed increasing number of decondensed sperm head inside. In fact, it was the only group that showed ≥10 of fused sperm. However, oocytes matured with LA at both concentrations mostly preserved the original pattern of the non-vitrified controls, with the highest percentages of fused sperm in the first range (1 to 3 fused spermatozoa with nuclear decondensation). Oocytes matured in 43 μM LA further preserved the original pattern of sperm fusion, concentrating the majority of oocytes in the first two categories. The behavior was inverse to that of the controls in terms of sperm fusion. Oocytes matured with LA decreased the number of fused sperm. Even when the following ranges (4 to 6 and 7 to 9 fused sperm heads) were registered, the percentages were lower than those of the vitrified control. On the other hand, vitrified control oocytes showed that as the range of fused spermatozoa increased, the amount of adhered spermatozoa increased markedly (mode values). In contrast, LA-matured and vitrified oocytes, regardless of LA concentration, showed a low and stable number of adhered sperm (Figure 7C). Across the ranges of fused sperm, LA-matured oocytes also better preserved the adhesion pattern compared to the control.

## 4 Discussion

Obtaining viable embryos from cryopreserved oocytes has been a challenging process in most mammals. Advances in cryopreservation techniques, such as vitrification, and a deeper understanding of the biology of oocytes undergoing this procedure are contributing to improve success rates. However, in domestic species, the wide variety of vitrification media and cryo-devices currently available reflects a lack of standardization as well as a lack of validated protocols. Cryopreservation is known to affect the membrane integrity of oocytes. Combination of FITC-Annexin V and PI allows to identify cells undergoing apoptosis or necrosis (37, 38). In this study, exposure to CPAs or vitrification did not increase the externalization of PS at the membrane of bovine oocytes, consistent with previous observations in porcine (39, 40). However, the loss of membrane integrity we observed after vitrification of oocytes matured *in vitro* in the presence of serum and vitrified in a tubular device reveals a reduced cryotolerance of these oocytes compared to those matured under serum-free conditions. Excessive incorporation of serum lipids may explain this effect, in part by increasing susceptibility to oxidative stress, particularly lipoperoxidation (41). Under high lipid peroxidation rates the extent of oxidative damage overwhelms repair capacity, and the cells may induce apoptosis or necrosis programmed cell death (42). In this respect, the oocytes matured in EGF-HA compared to those matured in FSH-FBS, exhibited a more favorable outcome to vitrification in terms of membrane integrity, and to some extent, in caspase activation. Moreover, the lower levels of caspase activation and membrane integrity loss in oocytes matured under FBS-free conditions and vitrified on a surface device, compared to those vitrified in a tubular device, demonstrate that minimum-volume devices and optimized maturing conditions favor survival, particularly of oocytes. After immersing this surface cryo-device in liquid nitrogen, the strip can be covered with a plastic cap to protect it during storage. However, this is a truly open system, meaning the sample is directly exposed to liquid nitrogen. As a consequence, high cooling and warming rates can be achieved. The last years, attention has focused on warming, which was recognized as more critical than vitrification (43, 44). A particularly high warming rate protects the cells by impeding the recrystallization of small intracellular ice crystals formed during cooling (43). The reported cooling and warming rates for OPS are 20,000°C/min (6), while a warming rate of 117,000°C/min was experimentally measured for a surface device such as Cryotop® (44). More recently, the same warming rate was estimated for Cryotop® based on mathematical modeling of heat transfer during warming of vitrified samples (45).

Despite these advances in cryo-devices, exposure of oocytes to anisotonic solutions during vitrification and warming induces osmotic stress and abrupt changes in cell volume. Additionally, cooling leads to membrane phase transitions from a fluid to an ordered state as the temperature is reduced below the membrane’s transition temperature (46, 47). Modulation of plasma membrane lipids by supplementing culture media with sterols or unsaturated fatty acids is a strategy used to minimize membrane phase transitions and stabilize the membrane at low temperatures. Membrane incorporation of different classes of sterols has been widely explored in the male gamete of various species to improve post-cryopreservation survival (48–51). Although both approaches have been addressed in bovine oocytes, greater progress has been made in understanding the effects of cholesterol incorporation into the membrane (31, 52). On the other hand, unsaturated fatty acids increase membrane fluidity by introducing double bonds into the acyl chains of phospholipids, a strategy initially used in bovine oocyte and embryo freezing (16, 17). In the present study, *in situ* detection of activated caspases demonstrated that the incorporation of LA into the bovine oocyte membrane, when supplemented in the maturation medium at 43 μM, does not affect the apoptotic status of the oocytes after exposure to CPAs or vitrification. Similar results were observed regarding membrane integrity. On the contrary, when supplemented at 100 μM, it is clear that an abnormal level of LA at the oocyte membrane or as free fatty acid negatively affects oocyte cryo-survival by inducing apoptosis and secondary necrosis. This effect observed after vitrification was also seen in a considerable proportion of non-vitrified oocytes exposed to the vitrification and warming solutions, indicating that these oocytes were unable to tolerate exposure to CPAs.

Previously, we showed that the composition of the maturation medium influences the meiotic and developmental competence of bovine oocytes by modifying their proteome (9). The maternal contribution of transcripts and proteins to the zygote is determining for the transition from oocyte to embryo, mainly considering that transcriptional activity remains inactive until the embryonic genome is activated (53). The transcription factor OCT4, also called POU5F1, acts by binding to a specific octameric sequence in target genes to activate or repress gene expression (24). It is well-known the role of OCT4 in maintaining pluripotency in the early embryo (24). In bovines, embryonic OCT4 is required for the expression of NANOG in the blastocyst and for expanded blastocyst formation (23, 54). On the other hand, maternal OCT4 has been identified as a regulator of oocyte developmental competence, establishing a transcriptional network from oogenesis (25, 26). Oct4 is not expressed in mouse developmentally incompetent MII oocytes, which accounts for the upregulation of a group of Oct4-regulated genes involved in the induction of mitochondrial dysfunction and apoptosis (27). In this study, we report for the first time that *in vitro* matured bovine oocytes exhibit two distinct patterns of OCT4 localization: one diffuse, with a homogeneous dispersed distribution throughout the cytoplasm, and another peri-metaphasic, characterized by moderate fluorescence in the cytoplasm and intense fluorescence surrounding the metaphase plate of the oocyte. In mouse, Oct4 was only detected in developmentally competent MII oocytes in the area around the MII plate (53). In bovines, OCT4 was detected by immunofluorescence mainly in the nucleus of oocytes at the germinal vesicle stage and to a lesser extent in the cytoplasm of immature and MII oocytes (55). The differences we found in the proportion of oocytes showing one expression pattern or another would be related to the quality of the oocytes and to the effect of the vitrification process. Thus, non-vitrified (fresh) oocytes present a higher proportion of the peri-metaphasic pattern compared to vitrified ones, an optimized localization for a transcription factor. Conversely, the diffuse pattern mostly observed after vitrification evidences the impact of this cryopreservation method on subcellular components. In this regard, all C+ oocytes after vitrification showed the diffuse pattern of OCT4. However, not all oocytes with a diffuse OCT4 pattern were C+. In this scenario, the addition of LA during oocyte maturation and its incorporation into the plasma membrane (18) was not sufficient to counteract the effects of vitrification on OCT4 localization and preserve OCT4 patterns. Furthermore, the increase in the diffuse pattern of OCT4 after CPA-exposure of oocytes matured in 100 μM LA revealed the importance of monitoring the intrinsic toxicity caused by CPAs, consistent with the previous effects we found at this high concentration of LA.

As noted, a major site of injury during oocyte cryopreservation is the plasma membrane. In this respect, a role of LA could be expected in events directly related to the plasma membrane, such as the competence of vitrified oocytes for fertilization—a parameter of functional survival, which refers to the ability of a cell not only to maintain morphological integrity after vitrification, but also to remain biologically competent (44). In this study, we used ZP-free oocytes co-incubated with sperm as a model to evaluate the membrane adhesion and fusion mechanisms in oocytes with higher levels of lipid unsaturation in the membrane. Within empirical limits, nuclear decondensation of spermatozoa inside oocytes was considered an indicator of functional fusion mechanisms. Under control conditions, analysis of sperm-oocyte fusion revealed that the absence of the ZP did not lead to high polyspermy (only 1 to 3 fused sperm per oocyte were found), suggesting an effective membrane polyspermy block in bovines (56, 57). *In vitro* maturation in the presence of LA did not alter the oocyte membrane ability to bind sperm, as approximately 70% of oocytes in all conditions showed sperm-egg binding. Regardless of LA concentration, fertilization rates were higher in LA-matured oocytes compared to controls, where approximately half of the LA-matured oocytes remained ‘normospermic’ (1 to 3 fused sperm per oocyte) and exhibited a low number of membrane-bound sperm. Those oocytes matured in LA that showed higher sperm penetration and a higher number of membrane-bound sperm compared to control oocytes or normospermic oocytes matured in LA, appear to have a weaker membrane block to polyspermy at the level of sperm-egg binding. Consistent with this, the membrane block to polyspermy in mice appears to occur at the level of sperm-egg binding, as fertilized eggs have been observed to lose the egg membrane protein Juno, which functions as a receptor for the sperm protein IZUMO1 (58). Post-fertilization reduction in the capacity to support sperm binding was less robust in part of the non-vitrified oocytes matured in LA.

When the oocytes were vitrified, the behavior described above was drastically modified. After vitrification, fertilized control oocytes showed augmented sperm penetration, which was increasingly evident across all the categories of fused sperm (until reaching ~40% of the oocytes with ≥ 10 fused sperm). Likewise, the number of membrane-bound sperm increased concomitantly with the increasing categories of fused spermatozoa, meaning that membrane block to polyspermy at the level of sperm-egg binding was altered in control oocytes after vitrification. On the contrary, oocytes matured with LA at both concentrations exhibited the inverse behavior in terms of sperm adhesion and fusion. Particularly, oocytes matured in 43 μM LA better preserved the adhesion and fusion pattern of non-vitrified controls by concentrating the majority of oocytes in the first two categories of fused sperm and maintaining a low and stable number of membrane-bound sperm. In addition, while oocytes matured in 43 μM LA maintained a fertilization rate similar to controls, oocytes matured in 100 μM LA did not support this fertilization rate after vitrification. Overall, this implies that even when oocytes matured in 43 μM LA did not show an improvement in the fertilization rate after vitrification, prior incorporation of LA into the oocyte plasma membrane preserved a more typical pattern of sperm binding and fusion, maintaining both the ability of the oocyte membrane to bind sperm and undergo fusion and to block excessive polyspermy in a controlled manner.

Despite many advances in the field, the embryo development rates of bovine oocytes after vitrification remain low (59–61). Future research will focus on developing optimized fertilization and embryo culture conditions to support the metabolic requirements of vitrified bovine oocytes. Improving cryopreservation outcomes is particularly relevant given the growing interest in biobanks, including gamete (germplasm) and tissue cryobanks (62, 63), which aim not only to conserve genetic resources but also to improve the reproductive efficiency of livestock.

In conclusion, results from our study showed that *in vitro* maturation of bovine oocytes under serum-free conditions favors oocyte survival after vitrification, specifically in terms of membrane integrity. Analysis of oocyte viability and caspase activation demonstrated that survival is enhanced when oocytes are *in vitro* matured in a synthetic maturation medium based on EG-HA and vitrified using a surface device. The supplementation of this maturation medium with 43 μM LA did not compromise the viability or apoptotic status of oocytes after vitrification. Conversely, higher concentrations of LA (100 μM) resulted in a significant increase in apoptosis, along with a loss of oocyte membrane integrity following vitrification, rendering these oocytes inefficient at tolerating CPA exposure. For the first time we identified two distinct localization patterns of the transcription factor OCT4 in bovine matured oocytes: one diffuse, dispersed in the cytoplasm, and another peri-metaphasic. Vitrification altered the relative distribution of these patterns, with the diffuse pattern becoming predominant. The addition of LA, regardless of concentration, was not sufficient to mitigate the effects of vitrification on the distribution of OCT4 patterns. Unlike with 43 μM LA, monitoring of CPA toxicity evidenced an increase in the diffuse OCT4 pattern in non-vitrified oocytes matured with 100 μM. Finally, ZP-free oocytes used as a model to evaluate oocyte membrane competence to bind and fuse sperm revealed that oocytes matured in 43 μM LA better preserved the original adhesion and fusion pattern after vitrification. Although fertilization rate was not improved after vitrification at this LA concentration, prior incorporation of LA into the membrane contributed to preserving the inherent ability of these oocytes to properly interact with sperm, maintaining membrane functionality. These effects were not manifested at higher concentrations of LA (100 μM). Taken together, findings from our study demonstrate that minimum-volume devices, particularly surface cryo-devices, and optimized maturation conditions (serum-free media combined with membrane-modulating lipids, such as 43 μM LA) enhance oocyte intrinsic quality, survival, and fusion competence after vitrification, crucial features for improving fertilization success of these oocytes.

## Data Availability

The datasets presented in this study can be found in online repositories. The names of the repository/repositories and accession number(s) can be found below: https://repositorio.inta.gob.ar/xmlui/handle/20.500.12123/22579 (part of the original contributions presented in the study are publicly available).

## In memoriam

The authors dedicate this manuscript to Prof. Dr. Ricardo H. Alberio, *in memoriam*.
